# Profile of Polyphenols, Fatty Acids, and Terpenes in Henola Hemp Seeds Depending on the Method of Fertilization

**DOI:** 10.3390/molecules29174178

**Published:** 2024-09-03

**Authors:** Anna Przybylska-Balcerek, Jakub Frankowski, Małgorzata Graczyk, Grażyna Niedziela, Dominika Sieracka, Stanisław Wacławek, Tereza Hulswit Sázavská, Maciej Buśko, Lidia Szwajkowska-Michałek, Kinga Stuper-Szablewska

**Affiliations:** 1 Department of Chemistry, Faculty of Forestry and Wood Technology, Poznań University of Life Sciences, 60-628 Poznań, Poland; maciej.busko@up.poznan.pl (M.B.); lidia.szwajkowska@up.poznan.pl (L.S.-M.); kinga.stuper@up.poznan.pl (K.S.-S.); 2 Department of Bioeconomy, Institute of Natural Fibres and Medicinal Plants—National Research Institute, Wojska Polskiego 71b, 60-630 Poznań, Poland; jakub.frankowski@iwnirz.pl (J.F.); dominika.sieracka@iwnirz.pl (D.S.); 3 Department of Mathematical and Statistical Methods, Poznan University of Life Sciences, 60-656 Poznań, Poland; malgorzata.graczyk@up.poznan.pl (M.G.); grazyna.niedziela@up.poznan.pl (G.N.); 4 Faculty of Mechatronics, Informatics and Interdisciplinary Studies, Technical University of Liberec, 461 17 Liberec, Czech Republic; stanislaw.waclawek@tul.cz; 5 Institute for Nanomaterials, Advanced Technologies and Innovation, Technical University of Liberec, 461 17 Liberec, Czech Republic; tereza.sazavska@tul.cz

**Keywords:** hemp seeds, bioactive compounds, polyphenols, phenolic acids, flavonoids, fatty acids, volatile organic compounds, sterols, carotenoids, fertilization

## Abstract

Botanical varieties of hemp differ in chemical composition, plant morphology, agronomy, and industrial suitability. Hemp is popular for cultivation for the production of cannabinoid oil, fiber production, biomass, etc. The fertilization process is one of the most important factors affecting the plant, both its condition and chemical composition. So far, research has been carried out proving that hemp is a valuable source of, among others: fatty acids, amino acids, acids, vitamins, numerous micro- and macroelements, and antioxidant compounds. In this experiment, it was decided to check the possibility of harvesting hemp panicles twice in one year. The purpose of this treatment is to use one plant to produce cannabidiol oil and grain. The main aim of the research was to determine bioactive compounds in hemp seeds and to determine whether the cultivation method affects their content and quantity. Based on the research conducted, it was observed that hemp can be grown in two directions at the same time and harvested twice because its health-promoting properties do not lose their value. It was found that regardless of whether hemp is grown solely for seeds or to obtain essential oils and then seeds, the type of fertilization does not affect the content of phenolic acids (e.g., syringic acid: 69.69–75.14 μg/100 g, vanillic acid: 1.47–1.63 μg/100 g). Based on the conducted research, it was found that essential oils can be obtained from one plant in the summer and seeds from Henola hemp cultivation in the autumn, because such a treatment does not affect the content of the discussed compounds.

## 1. Introduction

Hemp (*Cannabis sativa* L.) is an ancient crop with many uses. The first mention of the use of hemp can be found in a Chinese manuscript from the 28th century BC. It is believed that hemp was the oldest fiber plant cultivated by the Japanese, Mongols, and Tatars. The Cannabis species probably comes from Central Asia, from where it spread to East and South Asia and west to Europe. Currently, hemp is cultivated in approximately 30 countries in Europe, Asia, and North and South America [1]. Production includes both hemp seeds and crop waste. The composition of seeds may change depending on the variety, harvesting time, climatic conditions, and local agronomic factors [2]. Hemp (*Cannabis sativa* L.) is an annual, dioecious plant belonging to the Rosales order of the hemp family (Cannabaceae) [3]. Botanical varieties differ in chemical composition, plant morphology, agronomy, and suitability for industrial (industrial hemp) and pharmaceutical (medical hemp) processing [4].

The fertilization process is one of the most important factors affecting the plant, both its condition and chemical composition. In the case of hemp, as in the case of other oilplant oilseed plants, the supply of the right amount of nutrients can affect the composition of oil. Not only the profile of fatty acids but also the profile of volatile compounds responded with the aroma of the oil. These are essential factors responsible for the final quality of the oil, and they also play an important role in communication between animals and plants, including acting as attractants for pollinating insects or protecting against predators. 

So far, numerous studies have been carried out on the composition of both the grain itself and the products made from it, and it has been confirmed that this raw material is a very valuable source of many nutrients, such as fatty acids (constitute energy and reserve material, and also perform protective functions and influence the regulation of proper body temperature), amino acids (are responsible for the body’s hormonal balance and cell regeneration, and also participate in the metabolism of carbohydrates and fats), vitamins, numerous micro- and macroelements and antioxidant compounds (neutralize free oxygen radicals, delaying the aging process and the development of diseases, preclinical study showed that plant-derived chemicals like alkaloids, terpenes, flavonoids, phenolic acids, lignans, cinnamates, and saponins possess anxiolytic properties using various animal models of anxiety-like behavior) [5,6,7,8,9].

In the last decade, cultivation for the production of cannabinoid oil has also become very popular. For the production of cannabidiol oil, panicles of plants (the upper part where the flowers are located) are used, which should be harvested in full blooming. Every cultivation variation makes it possible to collect additional straw. Based on previous research where the positive effect of organic fertilization and increasing nitrogen doses has already been determined [10,11,12], in this case the effect of enriching the fertilizer with microelements on the size and quality of the hemp seed yield was analyzed. Moreover, in this experiment, it was decided to test the possibility of harvesting hemp from the same area in one year to obtain both panicles for the production of cannabidiol oil and grain. The main aim of the research was to determine bioactive compounds in hemp seeds and the effect of fertilization and cultivation methods on their content in the tested raw material.

## 2. Results and Discussion

### 2.1. Fatty Acids

In terms of industrial applications, fats are the most important component of seeds. The oil content in seeds, its composition, and its quality depend on environmental factors, such as cultivation area, agrotechnical treatments, variety, processing method, and storage conditions. In this study, the fatty acid profile of hemp seeds was investigated, with a particular focus on the presence of linoleic acid, α-linolenic acid, and γ-linolenic acid. Additionally, the study evaluated the omega-6 to omega-3 fatty acid ratio in various hemp seed variants. The study analyzed 15 different fatty acids present in hemp seeds. The study found that all tested variants of hemp seeds exhibited a ratio of omega-6 to omega-3 fatty acids of approximately 3:1. This ratio may vary depending on a number of factors, including the plant genotype and harvest year [13]. Irakli et al. (2019) showed that it can be 3.9 ÷ 5.5 [2], while Kiralan et al. (2010) recorded 2.8 ÷ 3.4 [14]. This ratio falls within the recommended range for a healthy diet and is considered favorable for maintaining optimal health. Among the identified fatty acids, linoleic acid showed the highest content in hemp seeds. The Henola 2 seed variant contained the most significant amount of linoleic acid, reaching 56.79%. The study revealed that α-linolenic acid was present in substantial quantities in the tested hemp seed variants. Specifically, the Henola 2-NPK+micro variant α-linolenic acid contents are 21.88%. α-linolenic acid is a precursor to other essential omega-3 fatty acids, such as EPA and DHA. From the profile of 15 acids, the presence of linoleic, α-linolenic, and γ-linolenic acids was found (Table 1). All tested seed variants are characterized by a ratio of omega-6 to omega-3 acids of about 3:1; the highest content of linoleic acid is omega-6 (Henola 2-NPK seeds: 56.79%; Henola 1-NPK: 55.83%), and the highest content of α-linolenic acid is omega-3 (Henola 2—NPK + micro: 21.88%; Henola 1—NPK + micro: 21.41%). In the literature on the subject, Kiralan et al. (2010) obtained similar results for hemp seeds from northwestern Turkey [14]. They determined linoleic acid at the level of 55.42%÷ 56.94%, α-linolenic acid-16.51% ÷ 20.40%. They confirmed the presence of small amounts of γ-linolenic acids (0.64% ÷ 1.10%) and stearic acids (0.34% ÷ 0.47%). Chen and Liu (2020) [15] and Kiralan et al. (2010) [14] report that the content of γ-linolenic acid is higher in grains grown in regions with a temperate and cold climate than in those grown in areas with a mild and warm climate. According to the above-mentioned authors, the content of oleic acid ranged from 11.40% to 15.88%.

**Figure 1:**
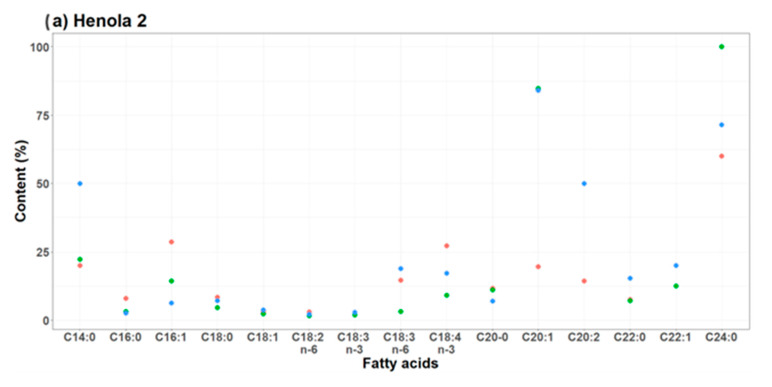

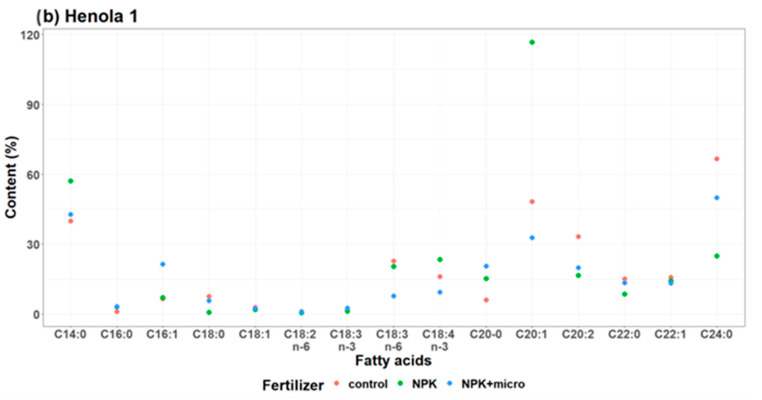
Coefficients of variation for various soil conditions: (**•**) Control, (**•**) NPK, (**•**) NPK+micro.

Based on the observed values of acid content in plants, coefficients of variation were determined for each of them. Their values are presented in Table 1 and in Figure 1. A coefficient value below 20 indicates little variability in the observed acid content in plants. Both in the case of Henola 1 and Henola 2, for acids C16:0, C16:1, C18:0, C18:1, C18:2 n−6, C18:3 n−6, C18:3 n−3, C18:4 n−3, C20:0, C22:1, C22:0, no significant variability was observed in their content in each of the studied groups of environmental conditions.

The dispersion of the C14:0 acid content increased in Henola 2 under NPK+micro fertilization conditions and in Henola 1 under NPK fertilization conditions.

In Henola 2, in the group of C20:1, C20:2 acids, the addition of fertilizer (both NPK and NPK+micro) resulted in an increase in the variation in their content values, while in Henola 1, the greatest increase in the variation in the C20:1 content was observed in NPK conditions. The variability of C24:0 acid content was significantly related to the plant. In Henola 2, the highest variability was observed in the case of NPK, and in Henola 1 in control conditions.

### 2.2. Profile of Phenolic Acids and Flavonoids

This paper presents the results of the analysis of the quantitative and qualitative profile of the polyphenols tested in hemp seeds and the presence of eight phenolic acids, with particular emphasis on syringic and caffeic acids, and three flavonoids. Table 2 presents the comprehensive analysis results, revealing the presence of eight different phenolic acids in hemp seeds, regardless of the purpose of the seeds and the method of fertilization. Among these phenolic acids, syringic acid has been consistently found to be the most abundant. For example, in the Henola 1-NPK variant, the content of syringic acid was 75.14 µg/100 g, which indicates its presence in hemp seeds. Another noteworthy finding from the data is the presence of caffeic acid in all tested cannabis seed variants. Caffeic acid is known for its potential health-promoting effects, which makes its presence in hemp seeds an encouraging discovery. The results of this analysis show that hemp seeds are a rich source of phenolic acids, with syringic acid and caffeic acid being the standout compounds. These results are consistent with previous research indicating the potential health benefits of these phenolic acids. Syringic acid has been linked to antioxidant and anti-inflammatory properties, which may contribute to the overall health-promoting effects of hemp seeds. In addition, the presence of caffeic acid is of interest for its well-known antioxidant activity, which can increase the nutritional value of hemp seeds. On the other hand, flavonoids are a group of bioactive compounds found in various plant species known for their potential health benefits and antioxidant properties. In this study, researchers analyzed the quantitative flavonoid profile in Henola seeds, focusing specifically on three compounds: rutin, naringenin, and quercetin (Table 3). The analysis showed significant differences in the content of flavonoids between the tested Henola variants. Among the three analyzed compounds, naringenin showed the highest concentration in all variants. It is worth noting that in Henola 2 seeds (NPK + micro), the content of naringenin was measured at an impressive level of 867.00 µg/100 g. This suggests that Henola 2 seeds have a significant presence of naringenin, which makes them an attractive potential source of this bioactive compound. Henola 2 seeds were found to contain at least 20 μg/100g more naringenin than Henola 1 seeds, regardless of fertilization variants. This indicates that the choice of seed variant can significantly affect the naringenin content, making Henola 2 the preferred choice for naringenin accumulation. In contrast to naringenin, the rutin content was approximately 35 μg/100g higher in Henola 1 seeds compared with Henola 2 seeds. This finding suggests that Henola 1 seeds may be a better option for those seeking a higher level of rutin. 

Hemp seeds are known to contain various bioactive compounds that can have different profiles in terms of type and quantity. These profiles can be influenced by factors such as the hemp variety, cultivation conditions (including fertilization and weather), and storage or processing methods [16,17,18]. However, it is important to note that not all variations are solely determined by agro–climatic factors [19,20,21]. In the literature, there is substantial information available on the composition of Henola hemp seeds. Phenolic compounds, fatty acids, and organic volatile compounds have been identified as the major bioactive compounds present in hemp seeds [22,23]. While the total phenolic compounds constitute around 0.8–1.5 of the dry matter, they are found in oils only in small amounts, typically ranging from 1.2 to 4.1 mg/100 g. Despite their low concentration, even these small amounts of phenolic compounds have a positive impact on the oxidative stability and ABTS anti-radical potential of hemp seeds [24,25,26]. Both during this and previous research, the primary polyphenols identified in the analyzed seeds were syringic and benzoic acids, along with naringenin [10]. Additionally, other researchers [27] have found that the phenolic profile includes lignan amides, phenolic amides, and various flavonoids such as isoflavones, flavonols, flavanones, and flavanols. Interestingly, it was observed that unshelled hemp seeds contain higher levels of polyphenols compared with shelled seeds, regardless of the agro–climatic conditions. Furthermore, the amount of biologically active compounds in hemp seeds was found to vary based on fertilization, with total phenolic content (TPC) ranging from 6.55 to 12.39 mg/gRUE and total flavonoid content (TFC) ranging from 2.52 to 4.74 mg/gRUE, as reported by other researchers [28]. Leonard et al. (2020) discovered that the total phenolic content in hemp seeds can also differ depending on the hemp variety and the fraction of the seed [29]. Moreover, it was observed in the literature that the content of total phenolic compounds in hemp seeds depended on their type and ranged from 831 mg/100 g dm to 1540 mg/100 g. These amounts are consistent with other literature data [30,31,32].

### 2.3. Terpenes Analysis 

#### 2.3.1. Volatile Organic Compounds—Monotermes and Sesquiterpenes

Terpenes are essential oils that do not have a psychoactive effect and do not belong to the cannabinoid group. However, they are secreted by trichomes-resin glands. The most expressive scents are emitted by unpollinated female flowers of hemp [5,6]. Terpenes are often underestimated, but in the presence of psychoactive cannabinoids, they provide positive, synergistic effects used in medicine as anti-anxiety substances [5]. Knowing the beneficial effects of terpenes on the human body, it is not surprising that they are also necessary to protect the cannabis plants themselves. Terpenes act as natural defense systems that attract pollinating organisms but protect against pests and bacteria while protecting against stressors in the environment. Therefore, some terpenes are extracted for use as all-natural insecticides and for use in other pest control methods [5,6]. The spectrum of terpenes contained in hemp is wide. Each type occurs in varying concentrations and with different frequencies, and they can be very volatile. In general, terpenes have a direct impact on the effectiveness of the action. One of the terpenes identified in hemp is limonene. It is characterized by a distinct citrus scent. This terpene is believed to have strong antifungal properties. Another terpene found in hemp is pinene. It is a two-ring terpene partially composed of a monoring terpene called limonene. Pinene is extracted and used for its anti-inflammatory properties. Mycerene, which is the most abundant terpene in hemp, is also worth mentioning. It is characterized by an earthy smell with notes of musk and cloves. Mycerene has anti-inflammatory properties and can be used as an analgesic [33]. However, the quality of hemp seeds is determined by the combination of terpenes (mono- and sesquiterpenes). The terpenes contained in hemp seeds have a bacteriostatic effect. This effect occurs mainly against Gram-positive bacteria (Staphylococcus and Streptococcus) [34]. Another important aspect is the content of the two terpenes, limonene and α-pinene, which act against repelling insects. During this study, an analysis of volatile organic compounds in *Canabis sativa* L. hemp seeds was performed. It was found that, regardless of the cultivation conditions, fertilization, and harvest stage, there were about 60 volatile organic compounds in each sample. However, the ones that most characterize hemp seeds are the seven monoterpenes and the nine sesquiterpenes described in Table 4. While the literature mentions a much larger number of compounds that determine the aroma of hemp seeds and hemp oil. Kaniewski et al. (2020) noted the presence of over 58 mono- and 38 sesquiterpenes (including: α-pinene, β-pinene, Δ3-carene, myrcene, limonene, β-phelandrene, cis-ocimene, trans-ocimene, α-terpinene, trans-α-bergamotene, β-caryophyllene, β-humulene, β-farnesene, β-selinene, seline-3.7(11)-diene), and others [35,36].

#### 2.3.2. Triterpenes and Tetraterpenes

During the conducted research, within a large group of terpenes, the presence of triterpenes, i.e., phytosterols, and tetraterpenes, i.e., carotenoids, was found (Table 3). In the literature on the subject, it is the commonly known sterol β-sitosterol [37]. Based on the results presented in Table 3, four phytosterols were detected among the triterpenes: campesterol, stigmasterol, β-sitosterol, and del-ta-5-avenasterol. It was found that the dominant phytosterol in Henola 2 seeds is β-sitosterol (NPK + microconditions = 179 mg/100 g). However, in previous studies, Frankowski et al. (2023) [10] found an approximately five lower content of these sterols. Based on the conducted research, the presence of tetraterpenes and three carotenoids, such as lutein, zeaxanthin, and beta-carotene, was also found (Table 3). It was found that the dominant carotenoid in Henola seeds (1 NPK + microconditions = 63.48 mg/kg) is beta-carotene. Similar observations were made by Frankowski et al. (2023) during his research (Table 5) [10].

**Figure 2:**
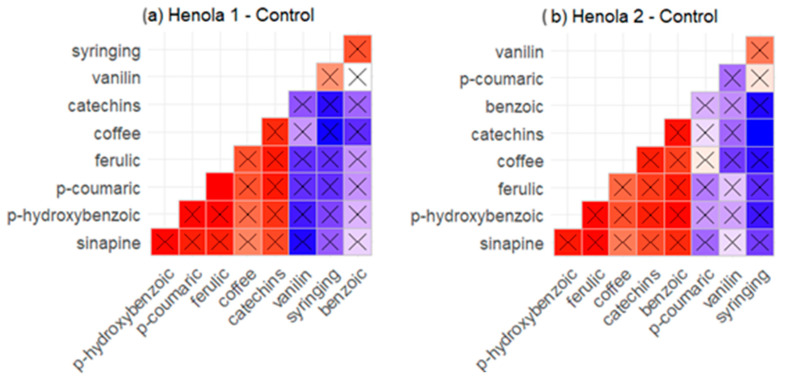

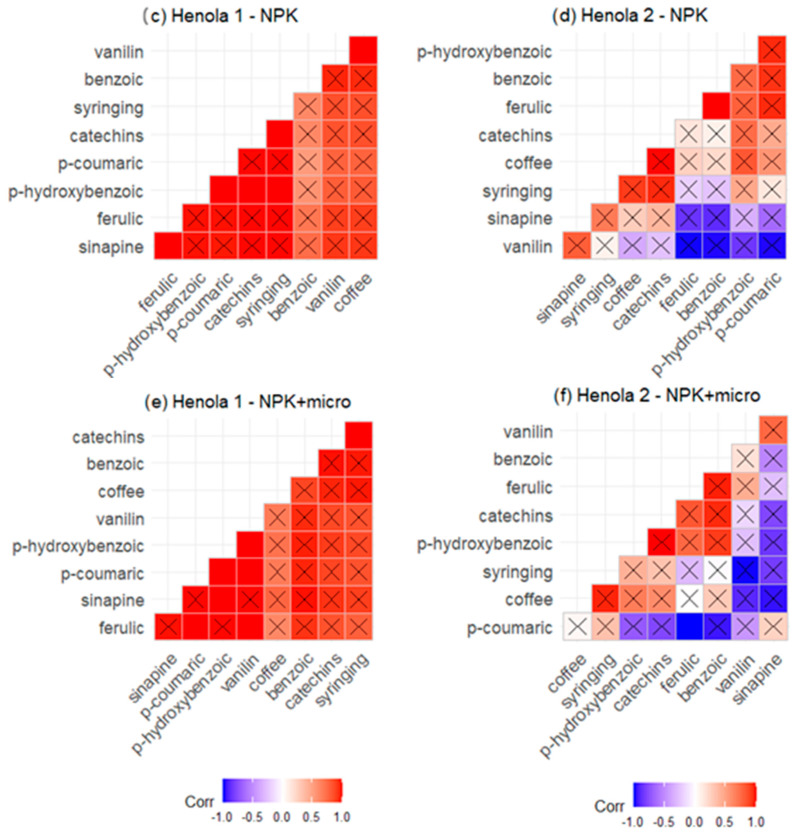
Visualization of the correlation matrix of phenolic acids in seeds for Henola 1 and Henola 2 in different soil conditions: (**a**) Henola 1—Control, (**b**) Henola 2—Control, (**c**) Henola 1—NPK, (**d**) Henola 2—NPK, (**e**) Henola 1—NPK+micro, (**f**) Henola 2—NPK+micro. X—statistically insignificant correlations.

Figure 2 visualizes the correlation matrices of phenolic acids in seeds under different soil conditions. There are both high correlations and relatively low correlations. Statistically insignificant correlations are plotted with a cross. On the other hand, among the flavonoids considered in the work, there is only a significant negative correlation between the content of naringenin and rutin under NPK conditions. Apart from this relationship, there are no significant correlations.

Under control conditions, only a significant positive correlation can be observed for p-coumaric and ferulic acids in Henola 1 plants. In contrast, a significant negative correlation is seen in Henola 2 only for catechins and syringing acids (Figure 2a,b).

In Henola 1 seeds in the conditions of enriched NPK soil, regardless of the content of microelements, the increase in the content of p-hydroxybenzoic acid has a positive effect on the content of p-coumaric. A similar tendency can be observed in the case of catechins and syringing acids (Figure 2c,e).

With NPK fertilization of the Henola 1 plant, the p-hydroxybenzoic acid content is significantly positively correlated with the content of catechins and syringing acids (Figure 2c). A similar trend can be observed in the case of vanillic and coffee, as well as sinapic and ferulic acids.

In Henola 2 seeds grown under NPK conditions, only a significant positive correlation was observed for benzoic and ferulic acids, Figure 2d).

In the conditions of fertilization with NPK+micro for Henola 1 seeds, the relationship between the content of vanillic acid and p-coumaric, ferulic, and p-hydroxybenzoic acid deserves attention (Figure 2e). With the increase in vanillic content, the content of ferulic, p-coumaric, and p-hydroxybenzoic acids increases, which is also correlated with the content of sinapic acid.

Only in plants growing in the best environmental conditions (i.e., NPK+micro), it was observed in Henola 1 seeds that with the increase in the content of p-coumaric acid, the content of ferulic acid increases, while in Henola 2 seeds, with the increase in the content of one of these acids, the content of the other decreases (Figure 2e,f).

**Figure 3:**
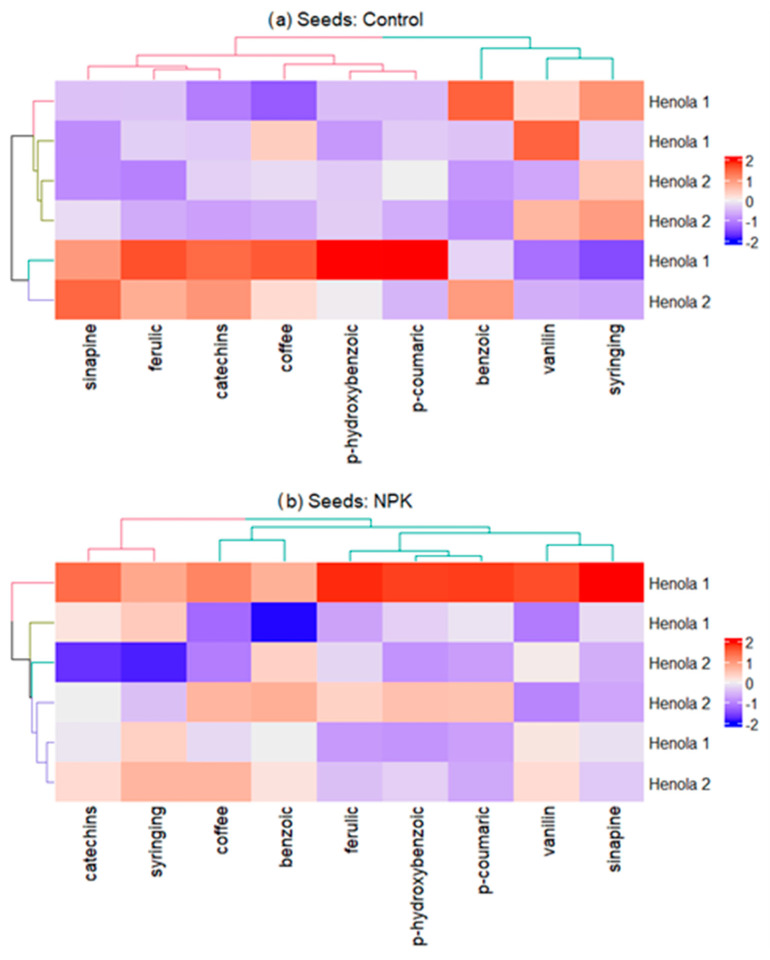

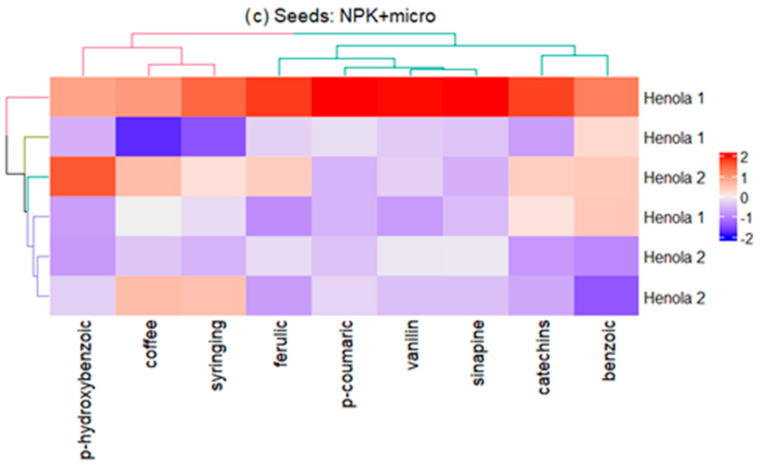
Heat map with dendrogram for the content of nine phenolic acids and two groups of Henola seeds in different soil conditions: (**a**) Control, (**b**) NPK, (**c**) NPK+micro.

Figure 3 presents a heat map with a dendrogram for the content of nine phenolic acids and two groups of seeds (Henola 1 and Henola 2) in three different soil conditions: Control, NPK, and NPK+micro. Analyzing all environmental conditions, due to the content of all phenolic acids, Henola 1 and Henola 2 do not form separate clusters. Under the conditions of NPK fertilization with possible micro enrichment, the content of ferulic, p-coumaric, vanillic, and sinapic acids is similar and significantly different from the content of syringing and benzoic acids (Figure 3b,c). Differences can be noted for catechins acid, whose content is similar to syringing in NPK conditions and to benzoic acid in NPK+micro conditions. In the conditions of fertilization with NPK+micro, the content of coffee and p-hydroxybenzoic acids is similar to the content of syringing, while only in the conditions of NPK, the content of coffee is comparable to the content of benzoic acid (Figure 3b,c). In the control conditions (Figure 3a), vanillic, syringing, and benzoic acids form the first cluster; the second cluster is coffee, p-hydroxybenzoic, p-coumaric, sinapic, ferulic, and catechins. Under the conditions of NPK fertilization (Figure 3b), catechins and syringing acids form the first cluster; the second includes coffee and benzoic acids; and the third is ferulic, p-coumaric, vanillic, sinapic, catechins, and benzoic. On the other hand, in plants growing in the best environmental conditions, NPK+micro (Figure 3c), p-hydroxybenzoic, coffee, and syringing acids include the first cluster, and the second one includes ferulic, p-coumaric, vanillic, and sinapic acids, and the third cluster contains catechins and benzoic acids.

**Figure 4:**
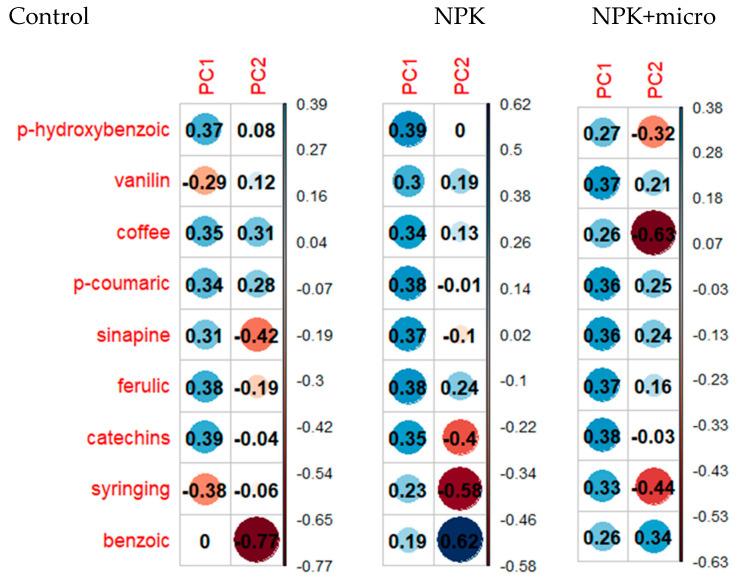
Contribution of individual phenolic acids to the formation of the first two principal components under different soil conditions: Control, NPK, NPK+micro.

Principal component analysis was carried out for the content of phenolic acids in hemp seeds grown under three different fertilization conditions in order to determine the similarities and differences in the quality of seeds from Henola 1 and Henola 2. Figure 4 presents the loads for the first two principal components for seeds grown in three different conditions, respectively. The loadings show us the contribution of individual base variables, i.e., phenolic acids, to the formation of principal components.

**Figure 5:**
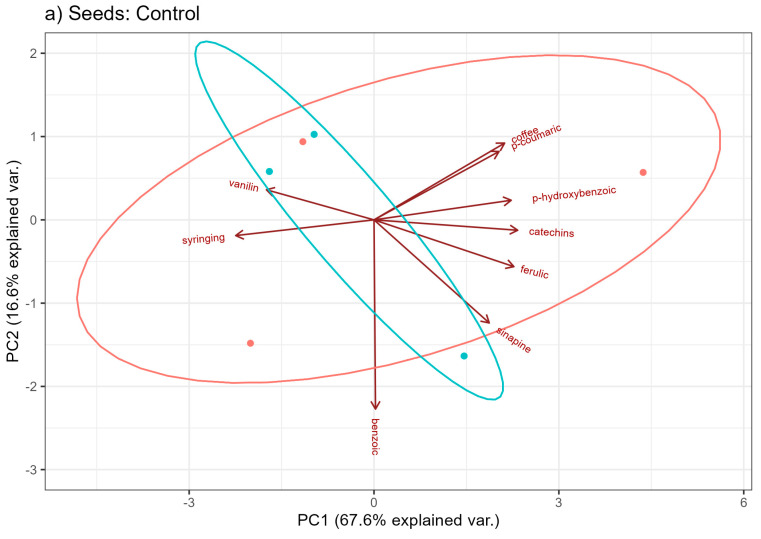

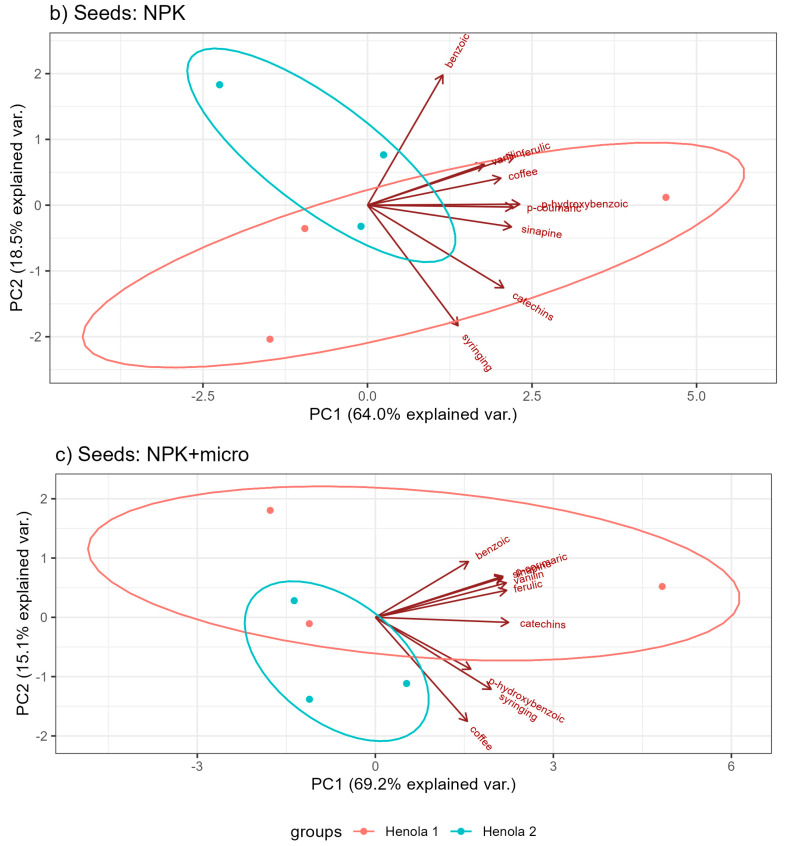
Biplot in different soil conditions: (**a**) Control, (**b**) NPK, (**c**) NPK+micro.

In turn, Figure 5a–c present individual observations and features (the content of phenolic acids) on the plane in the system of the first two main components in different fertilization conditions: Control, NPK, and NPK+micro. Under the control conditions (Figure 5a), the first two principal components explain over 84 of the total variability of the nine examined features. An analogous situation is visible when fertilizing with NPK+micro (Figure 5c). Here, too, the loss of information regarding the variability between the contents of phenolic acids at the transition from the nine-dimensional space of original variables to the space of the first two principal components is about 16. In Figure 5b, under the conditions of NPK fertilization, the two main components, which are a linear combination of the input hemp characteristics, explain over 82 of the total variability, i.e., the loss of information due to dimension reduction is about 18. Under the control conditions (Figure 4), the features most strongly correlated with the first principal component are the following acids: catechins, ferulic, syringing, and p-hydroxybenzoic. The most significant influences on the second main component are the acids: benzoic, sinapic, and coffee. When fertilizing with NPK, the features most strongly correlated with the first main component are the acids: p-hydroxybenzoic, ferulic, p-coumaric, and sinapic, while with the second main component are the acids: benzoic, syringing, and catechins.

Under NPK+micro conditions, catechins, ferulic, and vanillic acids significantly (positively) affect the value of the first component. The value of the second component is most positively influenced by benzoic acid, while the content of coffee, syringing, and p-hydroxybenzoic acids has a negative impact.

It can be observed that in all three conditions, the vast majority of acids have a positive effect on the first principal component. And on the second component, the greatest influences are negative (Figure 4 and Figure 5). It is also seen that catechins and ferulic acids have a large positive effect on the first principal component under all conditions.

In turn, syringing acid has a large negative effect on the first principal component under control conditions, but it also has a large negative effect on the second principal component already during fertilization. P-hydroxybenzoic acid has a large positive effect on the first component, but only under control and NPK conditions. On the other hand, benzoic acid clearly affects the form of the second main component in all three conditions of fertilization. Except that in control conditions and NPK, it has a negative effect, and in NPK+micro conditions it has a positive effect.

In Figure 5, no clear grouping of Henola 1 and Henola 2 can be observed.

Comparing Figure 5a–c, it can be concluded that the dispersion of individual observations is at a similar level in all fertilization conditions. The type of fertilization does not change the content of phenolic acids.

The similarities and differences in the content of phenolic acids in hemp seeds under different fertilization conditions were obtained by performing a principal component analysis for the content of the considered compounds in hemp seeds from Henola 1 and Henola 2.

**Figure 6:**
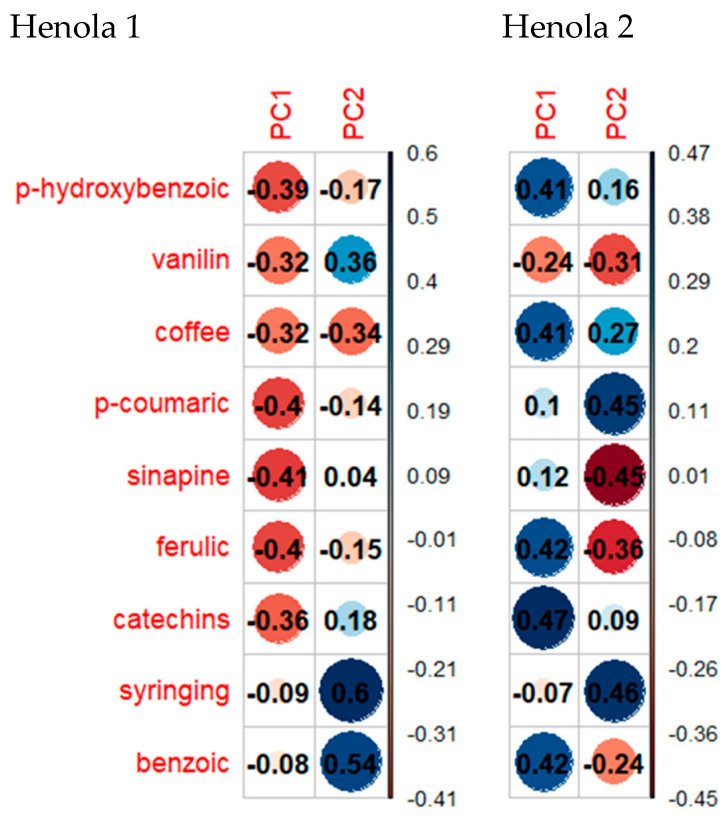
Contribution of individual phenolic acids in the formation of the first two principal components for Henola 1 and Henola 2 seeds.

The contribution to individual components, and thus the impact on the variability of the total acid content, is shown in Figure 6.

**Figure 7:**
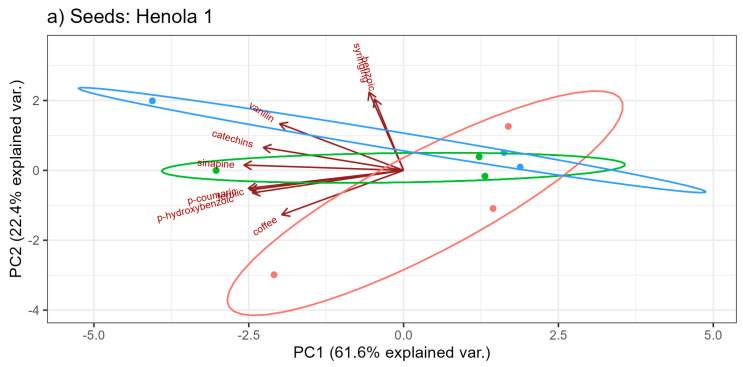

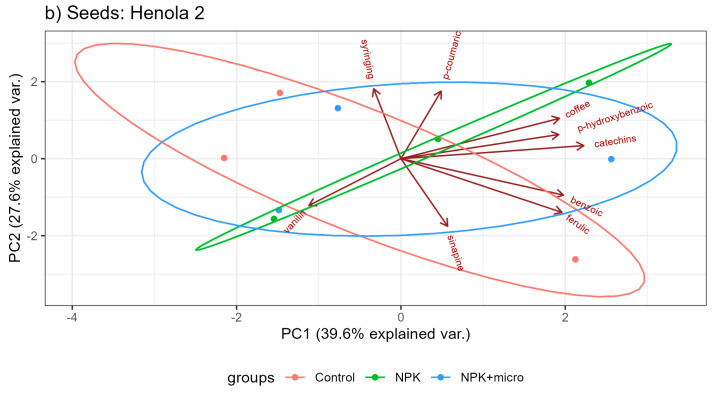
Biplot for the content of phenolic acids in seeds: (**a**) Henola 1, (**b**) Henola 2.

The relationships between the contents of phenolic acids expressed in terms of the first two principal components for Henola 1 and Henola 2 are shown in Figure 7.

In the experiment performed for Henola 1 seeds (Figure 7a), the first two principal components explain eighty-four of the total variability of the nine tested phenolic acids. In Figure 7b, in the experiment performed for Henola 2 seeds, two principal components, being a linear combination of the input hemp characteristics, explain slightly more than 67 of the total variability. So in this case, the information loss due to dimension reduction to two variables is higher than for Henola 1 seeds.

For Henola 1 (see Figure 7a), the features most strongly correlated with the first principal component are the following acids: sinapic, p-coumaric, ferulic, and p-hydroxybenzoic. Syringing and benzoic acids have the greatest influence on the second principal component. On the other hand, for Henola 2, catechins, benzoic, ferulic, coffee, and p-hydroxybenzoic acids significantly (positively) affect the value of the first component. The value of the second component is most influenced by syringing, sinapic, and p-coumaric.

It can be seen that in Henola 1 all acids have a negative effect on the first principal component, and in Henola 2 most acids have a positive effect on the first principal component (see Figure 6 and Figure 7).

In Figure 7, no clear grouping of fertilizations can be observed, respectively: Control, NPK, and NPK+micro. The type of fertilization does not change the content of phenolic acids.

Comparing Figure 7a,b, it can be concluded that the dispersion of individual observations is at a similar level for the content of acids in the seeds of Henola 1 and Henola 2. The method of cultivation does not change the content of phenolic acids.

Figure 7a shows that for Henola 1, there is a positive correlation between the variables, or it is at the level of zero. There are no negative correlations between phenolic acids. On the other hand, there are positive, negative, and zero relationships between the acid contents in Henola 2 seeds.

Principal component analysis was carried out for the content of flavonoids in hemp seeds grown under three different fertilization conditions in order to determine the similarities and differences in the quality of seeds from Henola 1 and Henola 2.

**Figure 8:**
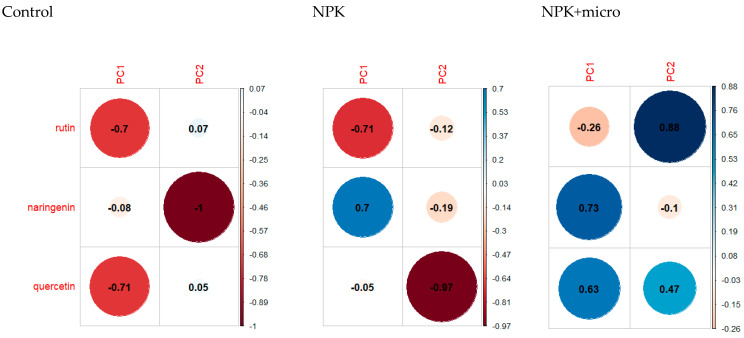
Contribution of individual flavonoids to the formation of the first two principal components under different fertilization conditions: Control, NPK, NPK+micro.

Figure 8 presents the loads for the first two principal components for seeds grown in three different conditions, respectively. The loads show us the contribution of individual base variables, i.e., flavonoids, to creating principal components.

**Figure 9:**
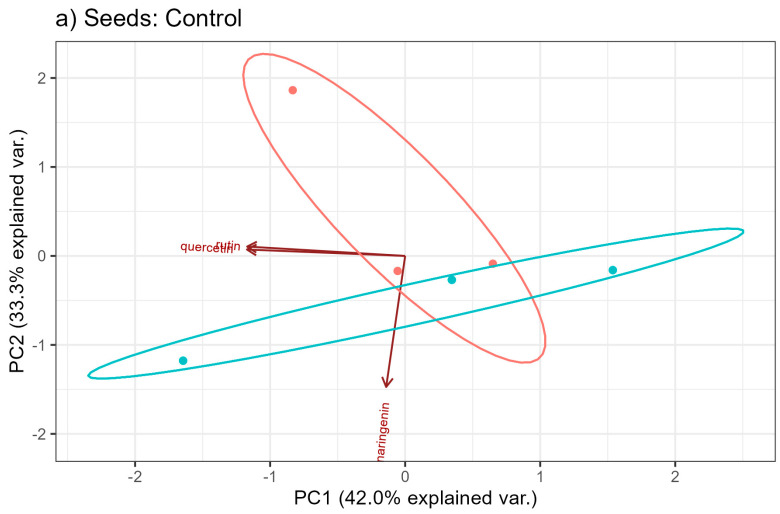

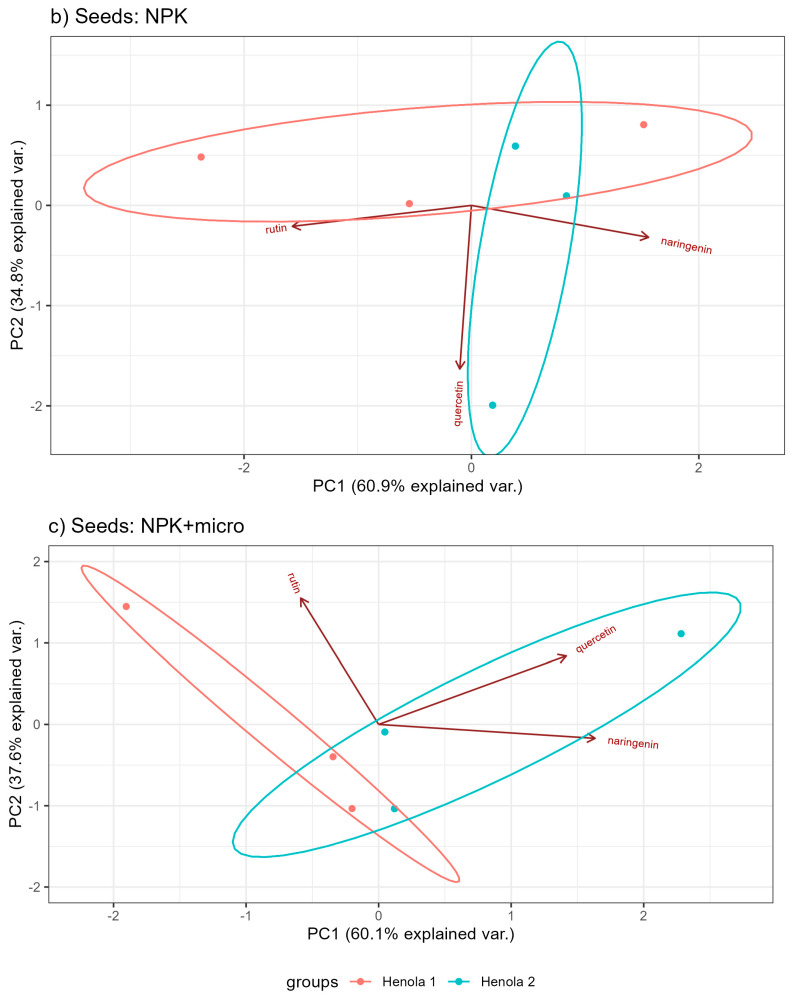
Biplot for the content of flavonoids in hemp seeds under different fertilization conditions: (**a**) Control, (**b**) NPK, (**c**) NPK+micro.

In order to graphically present the variability of flavonoid content, principal component analysis was used. Figure 9a–c show individual observations and features (the content of flavonoids) on the plane in the system of the first two principal components under different fertilization conditions: Control, NPK, and NPK+micro.

Under the control conditions (Figure 9a), the first two principal components explain over 75 of the total variability of the three examined features. In Figure 9b, under the conditions of NPK fertilization, the two main components, which are a linear combination of the input hemp characteristics, explain over 95 of the total variability, i.e., the loss of information due to dimension reduction is about 5. An analogous situation is visible when fertilizing with NPK+micro (see Figure 9c). Here, too, the loss of information is very small at the transition from the three-dimensional space of original variables to the space of the first two principal components and amounts to about 3.

Under the control conditions (see Figure 8 and Figure 9), the features most strongly correlated with the first principal component are rutin and quercetin. Naringenin has the greatest influence on the second principal component. When fertilizing with NPK, the features most strongly correlated with the first principal component are naringenin and rutin. However, the second main component is quercetin.

Under NPK+micro conditions, the flavonoids naringenin and quercetin significantly affect the value of the first component. Rutin has the greatest influence on the value of the second component.

It can be observed that in the control conditions, the content of flavonoids has a positive effect on the first principal component, and in the NPK+micro conditions the opposite is true (see Figure 8).

In Figure 9, no clear grouping of Henola 1 and Henola 2 can be observed.

Comparing Figure 9a–c, it can be concluded that the dispersion of individual observations is at a similar level in all fertilization conditions. The type of fertilization does not change the content of flavonoids.

Similarities and differences in flavonoid content in hemp seeds grown in different fertilization conditions were obtained by performing a principal component analysis for the content of the considered compounds in hemp seeds from Henola 1 and Henola 2.

**Figure 10:**
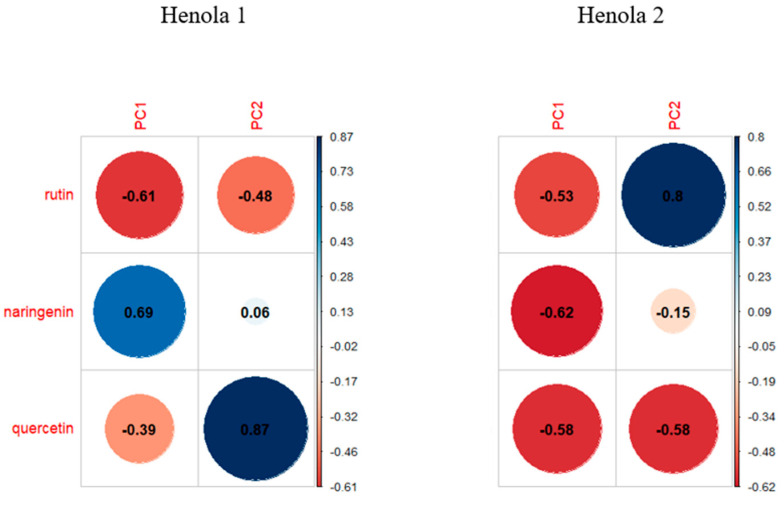
Contribution of individual flavonoids to the formation of the first two principal components for Henola 1 and Henola 2 seeds.

**Figure 11:**
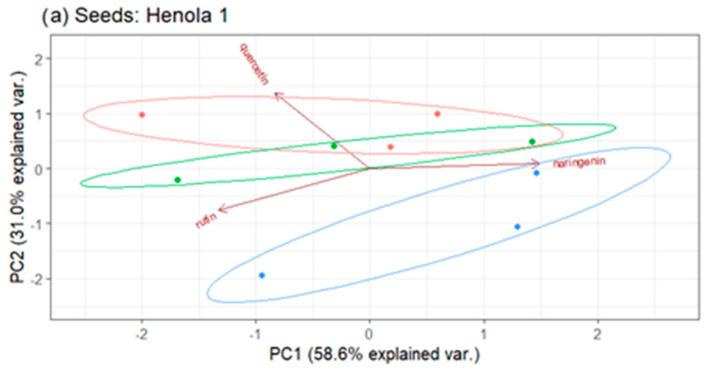

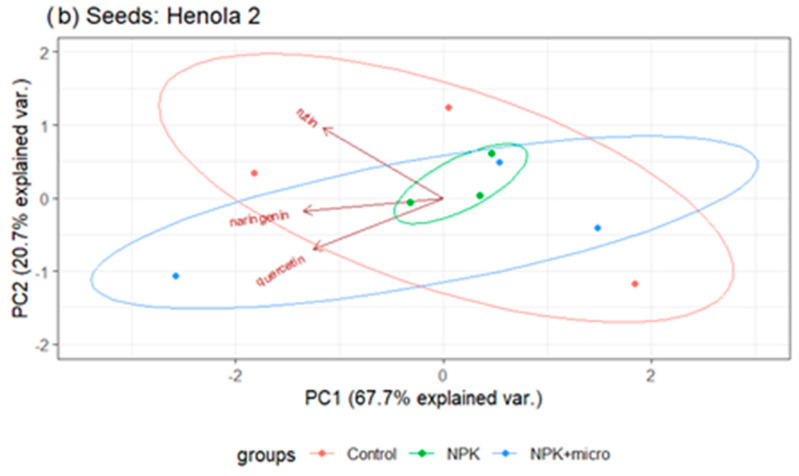
Biplot for flavonoid content in seeds (**a**) Henola 1, (**b**) Henola 2.

The contribution to individual components, and thus the impact on the variability of the overall flavonoid content, is shown in Figure 10. The relationships between the contents of flavonoids expressed in terms of the first two main components for Henola 1 and Henola 2 seeds are shown in Figure 11.

In the experiment performed for Henola 1 seeds (Figure 11a), the first two principal components explain over 89 of the total variability of the three flavonoids tested. In Figure 11b, in the experiment performed for Henola 2 seeds, two principal components, which are a linear combination of the input cannabis characteristics, explain slightly more than 88 of the total variability. The loss of information due to the reduction of the dimension to two variables is at a similar level for Henola 1 and Henola 2 seeds.

For Henola 1 seeds (see Figure 11a), the features most strongly correlated with the first principal component are naringenin and rutin. Quercetin and rutin have the greatest influence on the second principal component. On the other hand, for Henola 2, all three tested flavonoids significantly (negatively) affect the value of the first component. The value of the second component is most influenced by rutin and quercetin.

It can be seen that in Henola 1 and Henola 2 seeds, the content of naringenin has the greatest impact on the first principal component, but in Henola 1 it has a positive effect and in Henola 2 it has a negative effect. In turn, rutin and quercetin have the greatest influence on the second principal component, but these influences are opposite in Henola 1 and Henola 2 (see Figure 10 and Figure 11).

In Figure 11, no clear grouping of fertilizations can be observed, respectively: Control, NPK, and NPK+micro. The type of fertilization does not change the content of flavonoids.

Comparing Figure 11a,b, it can be concluded that the dispersion of individual observations is at a similar level for the content of flavonoids in the seeds of Henola 1 and Henola 2. The method of cultivation does not contribute to the change in the content of flavonoids.

The non-parametric Wilcoxon test was used to evaluate the differences between Henola 1 and Henola 2 seeds in the degree of phenolic acids and flavonoids content. The test was used with the assumption of significance at *p* < 0.05. Calculations were performed separately for each considered phenolic acid and flavonoids. Based on the obtained results (Table 6), it can be concluded that there are no differences between the distributions for Henola 1 and 2 seeds at the assumed significance level.

## 3. Materials and Methods

### 3.1. Methodology of Conducting the Experiment

The experiment was carried out at the Experimental Station of the Institute of Natural Fibers and Medicinal Plants—National Research Institute in Stary Sielec (51°39′36.15″ N 17°08′41.75″ E) in Poland in 2021–2022. Experimental fields of 30 m^2^ each were prepared in three repetitions.

Medium plowing was carried out in autumn, followed by dragging, cultivator, and harrows during the spring.

Experiment based on different variants of harvesting. In the first variant of the study (Henola 1), hemp seed was harvested in a standard way, once at full maturity, in autumn. The second variant (Henola 2) assumed a double harvest of hemp—the first in the summer, during which the flowering panicles of the plants were cut down for the purpose of extracting cannabidiol oil, and the second in the autumn, when the seeds were fully mature.

On each of the above variants, 3 types of fertilization were applied:

1. No mineral fertilization.
2. Basic mineral fertilization (in kg·ha^−1^): N—150; P_2_O_5_—40; K_2_O—80.
3. Full mineral fertilization (in kg·ha^−1^): 800 kg·ha^−1^ of Azofoska fertilizer: N—108.8; P_2_O_5_—51.2 K_2_O—152.8; MgO—36.0; SO_3_—184.0; B—0.36; Cu—1.44; Fe—1.36; Mn—2.16; Mo—0.32; Zn—0.36.

Nitrogen was supplied in the form of ammonium nitrate at a concentration of 117 kg·ha^−1^.

Directly before sowing the seeds, mineral fertilizers were applied and harrowed. 

Representative samples of grain were taken in three repetitions corresponding to every experimental variation. Manual harvesting of panicles, shade drying, deseeding, and further shade drying till approx. seeds humidity decreased to 15%. Storage conditions: paper bags in 10 °C. Processing timeline from harvesting panicles to preparing seeds for chemical analysis was approx. 3 weeks.

Samples were further analyzed in order to determine quantitative and qualitative composition of bioactive compounds.

### 3.2. Chemical Analyses

#### 3.2.1. Fatty Acids Analysis

The fatty acid content of the seeds was analyzed according to PN-EN ISO 11085:2015-10 [38].

In order to examine the degree of variation in the values of the variable determining the content of individual acids in plants, coefficients of variation were determined. A high value of this coefficient indicates a large diversity of the feature and proves the heterogeneity of the studied population. A low value indicates low variability of the feature and homogeneity of the studied population.

#### 3.2.2. Terpenes Analysis

##### Analysis of Volatile Organic Compounds Such as Monoterpenes and Sesquiterpenes

VOCs were extracted from the headspace of 20 mL vials containing 4000 g of hemp by means of the solid-phase microextraction (SPME) method using 200 mm of 53/30 mm DVB/Car-boxen/PDMS fiber. Extraction was carried out at 50 °C for 25 min. Afterwards, the SPME fiber was inserted into the injection GC port for thermal desorption at 260 °C for 2 min. For HS-SPME analysis, we used Thermo Scientific Trace 1310 gas chromatograph (Boston, MA, USA) and mass spectrophotometer TSQ 8000 Evo (Thermo Scientific, Boston, MA, USA). The column used for analyses was an Rxi-5MS (30 m × 0.25 mm × 0.25 µm) from Restek (Bellefonte, PA, USA). The method employed in this study adhered to rigorous parameters. The injector port temperature was maintained at 260 °C, ensuring efficient vaporization and injection of the sample. Helium 5.0, a commonly used carrier gas, was delivered at a constant flow of 1.0 mL/min, guaranteeing consistent sample movement through the column. The oven temperature, a crucial factor influencing separation, was meticulously controlled. Starting at 40 °C, it was ramped up at a rate of 10 °C/min to 180 °C and further increased to 260 °C at 40 °C/min, held steady for 5 min. These parameters, as reported by Przybylska-Balcerek et al. (2024), formed the foundation of the analysis [39].

Accurate quantification of VOCs was achieved by comparing the area under their total ion current peaks with a carefully calibrated internal standard, tridecane, injected into the samples. This method allowed for precise measurement, expressed as the ratio of areas, termed Relative Units (RU). To ensure identification fidelity, mass spectra of compounds were meticulously compared with those in the NBS 75K and NIST 98 libraries. Retention indices, calculated based on the chromatographic analysis of chain alkanes (C8–C20), served as critical data points for compound confirmation, enhancing the reliability of the findings.

The deconvolution and quantification of chromatograms using sophisticated software like AMDIS (Version 2.73) provided a detailed VOC profile of sorghum grain. The data obtained not only enriches our knowledge of sorghum chemistry but also has broader implications. Understanding the volatile composition of sorghum aids in its utilization in various sectors, from food and beverage industries to biofuel production. Additionally, this study’s rigorous methodology sets a benchmark for future research in VOC analysis, ensuring a higher degree of accuracy and reliability in similar studies.

##### Analysis of Triterpenes-Phytosterols

Samples containing 100 mg of ground grains were placed into 17 mL culture tubes, suspended in 2 mL of methanol, treated with 0.5 mL of 2 M aqueous sodium hydroxide, and tightly sealed. The culture tubes were then placed within 250 mL plastic bottles, tightly sealed, and placed inside a microwave oven (Model AVM 401/1 WH, Whirlpool, Stockholm, Sweden) operating at 2450 MHz and 900 W maximum output. Samples were irradiated (370 W) for 20 s and after about 5 min for an additional 20 s. After 15 min., the contents of culture tubes were neutralized with 1 M aqueous hydrochloric acid, 2 mL methanol were added, and extraction with pentane (3 × 4 mL) was carried out within the culture tubes. The combined pentane extracts were evaporated to dryness in a nitrogen stream. Before analysis, samples were dissolved in 4 mL of methanol, filtered through 13 mm syringe filters with a 0.5 mm pore diameter (Fluoropore Membrane Filters, Millipore, Carrigtwohill, Ireland), and evaporated to dryness in a N_2_ stream. The sample extract was dissolved in 1 mL of methanol, and 50 µL were analyzed by Acquity H class UPLC system equipped with a Waters Acquity PDA detector (Waters, Milford, MA, USA). Chromatographic separation was performed on an Acquity UPLC^®^ BEH C18 column (100 mm × 2.1 mm, particle size 1.7 μm) (Waters, Wexford, Ireland) eluted with methanol/acetonitrile/water (85:10:5) at a flow rate of 0.4 mL/min. Sterols were detected using a Waters Acquity PDA detector (Waters, USA) set at 282 nm [40,41].

##### Analysis of Tetraterpenes-Carotenoids

Carotenoid isolation and quantification were performed by Acquity UPLC (Waters, USA) in grain samples by the saponification method. Carotenoids extracts were obtained from ground beans (0.4 mg), which were triturated with a mixture of acetone and petroleum ether (1:1). Then, after separation of the plant tissue, the acetone and the hydrophilic fraction were removed from the extract by washing with water. As a result, the ether extract was obtained with a mixture of carotenoid pigments. The extract thus prepared was concentrated in a vacuum evaporator at 35 °C until an oily residue was obtained, then digested in 2 mL of methanol (Merck, Rahway, NJ, USA) and subjected to chromatographic analysis. Total carotenoids determined using Acquity UPLC (Waters, USA) with a Waters Acquity PDA detector (Waters, USA). Chromatographic separation was performed on an Acquity UPLC^®^ BEH C18 column (100 mm × 2.1 mm, particle size 1.7 μm) (Waters, Ireland). Elution was carried out using solvent A—methanol, B-water, and tert-butyl methyl ether (TBME). A gradient was applied at a flow of 0.4 mL/min. The column and samples were thermostated; the column temperature was 30 °C, the test temperature was 10 °C. During the analysis, the solutions were degassed in a Waters device. The injection volume was 10 μL. The registration was carried out at a wavelength λ = 445 nm. [42,43].

##### Analysis of Phenolic Acids and Flavonoids

Samples for analyses were weighed at 0.20 g. They were placed in sealed 17 mL culture test tubes, where first alkaline and then acid hydrolysis were run. In order to run alkaline hydrolysis, 1 mL distilled water and 4 mL 2 M aqueous sodium hydroxide were added to test tubes. Tightly sealed test tubes were heated in a water bath at 95 °C for 30 min. After cooling (approx. 20 min), test tubes were neutralized with 2 mL 6 M aqueous hydrochloric acid solution (pH 2). Next, samples were cooled in water with ice. Flavonoids were extracted from the inorganic phase using diethyl ether (2 × 2 mL). Formed ether extracts were continuously transferred to 8 mL vials. Next, acid hydrolysis was run. For this purpose, the aqueous phase was supplemented with 3 mL 6 M aqueous hydrochloric acid solution. Tightly sealed test tubes were heated in a water bath at 95 °C for 30 min. After being cooled in water with ice, the samples were extracted with diethyl ether (2 × 2 mL). Produced ether extracts were continuously transferred to 8 mL vials, after which they were evaporated to dryness in a stream of nitrogen. Prior to analysis, samples were dissolved in 1 mL methanol. Analysis was performed using an Aquity H class UPLC system equipped with a Waters Acquity PDA detector (Waters, USA). Chromatographic separation was performed on an Acquity UPLC^®^ BEH C18 column (100 mm × 2.1 mm, particle size 1.7 μm) (Waters, Ireland). The elution was carried out gradient using the following mobile phase composition: A: acetonitryl with 0.1 formic acid; B: 1 aqueous formic acid mixture (pH 2). Concentrations of flavonoids were determined using an internal standard at wavelengths λ = 320 nm. Concentrations of phenolic acids were determined using an interna standard at wavelengths λ = 280 nm. Compounds were identified based on a comparison of the retention time of the analyzed peak with the retention time of the standard and by adding a specific amount of the standard to the analyzed samples and a repeated analysis. Detection level is 1 μg/g. Retention times of assayed acids are as follows: kaempferol 6.11 min, gallic acid 8.85 min, vanillic 9.71 min, luteolin 11.89 min, protocatechuic acid 12.23 min, vanillic acid 14.19 min, apigenin 16.43 min, catechin 18.09 min, 4-hydroxybenzoic acid 19.46 min, chlorogenic acid 21.56 min, caffeic acid 26.19 min, syringic acid 28.05 min, naringenin 31.22 min, vitexin 35.41 min, rutin 38.11 min, quercetin 39.58 min, p-coumaric acid 40.20 min, ferulic acid 46.20 min, sinapic acid 48.00 min, and t-cynnamic acid 52.40 min, respectively. Recovery rates for the analyzed phenolic compounds were as follows: caempferol 86 ± 5.3, gallic acid 92 ± 4.4, vanillic 79 ± 8.5, luteolin 96 ± 2.7, protocatechuic acid 90 ± 4.8, vanillic acid 88 ± 5.1, apigenin 93 ± 3.8, catechin 89 ± 5.7, 4-hydroxybenzoic acid 96 ± 3.78, chlorogenic acid 92 ± 2.8, caffeic acid 86 ± 6.7, syringic acid 94 ± 3.9, naringenin 88 ± 4.8, vitexin 95 ± 3.8, rutin 93 ± 4.9, quercetin 97 ± 1.9, p-coumaric acid 89 ± 3.6, ferulic acid 91 ± 4.9, sinapic acid 94 ± 5.1 and t-cynnamic acid 97 ± 2.9 [42,44].

#### 3.2.3. Statistical Methods

As part of the research, the seeds of the *Cannabis sativa* L. Henola variety were analyzed. The experiment concerned a plant grown only for seeds (Henola 1), cultivated to obtain essential oils, and then for seeds as seed (Henola 2). The research was carried out on two variants (depending on the intended use), each cultivated in three different series (depending on fertilization). Each of these series was fertilized accordingly: in the first case no fertilization was performed (Control), in the second case only NPK (NPK) was fertilized, and in the third case NPK and micro (NPK + micro). Each sample was analyzed in three replicates. Statistical analysis includes the determination of correlation coefficients between phenolic acids and between flavonoids in seeds. They define the relationships between individual variables. First of all, the dependencies that are statistically significant were indicated. The analysis also uses cluster analysis methods that allow for grouping the examined objects according to their similarity hierarchy. The results are presented in the form of a heat map with dendrograms. In turn, in order to reduce the number of variables and detect regularities between the examined features, a popular technique was used—principal component analysis (PCA). There are large differences in the scale of the data; therefore, before starting this analysis, data scaling [13] was used, consisting of calculating the quotient of the difference of a single measurement and the mean by the standard deviation in the sample. Thanks to it, it was possible to replace the original variables with new variables (components), mutually independent, which successively explain a specific part of the variance of the initial variables. On the basis of the scree chart, such a number of components was extracted so that the percentage of explained variance was sufficient. In the system of the first two components, the vectors of the examined hemp traits are presented. The longer the vector representing the given features, the greater the share of this variable in the values of the first two components. The direction of the arrows shows the influence of these features on the first and second components, respectively. The angle of intersection of the vectors is proportional to the relationship between the features. The points, on the other hand, represent the individual observations in the new layout. This analysis allowed us to determine the effect of fertilization on the chemical composition of seeds and to compare the properties of Henola 1 and Henola 2 seeds. Finally, a non-parametric Wilcoxon test was performed to check whether Henola seeds 1 and 2 came from the same distribution. All statistical analyses were performed using R 4.3.0 software [R Core Team, 2023]. The drawings in this document were created using the R ggplot2 (version 3.4.2), ggcorrplot (version 0.1.4), ComplexHeatmap (version 2.16.0), corrplot (version 0.92) packages.

## 4. Conclusions

This research concerned a novel harvest of hemp. They consisted of the possibility of harvesting twice from the same area during one year of cultivation. A crop has been proposed that makes it possible to harvest from one plant twice during one year of cultivation in order to obtain material for the production of cannabidiol oil and seeds. The main aim of the research was to determine bioactive compounds in hemp seeds and to determine whether the cultivation method affects their content and quantity. Based on the conducted research, it was observed that regardless of environmental conditions, the Henola 1 and Henola 2 groups do not form separate groups. The lack of clear grouping and the lack of differences in the content of the considered compounds in the mentioned experiments compared with Henola 1 and Henola 2 allow us to conclude that growing hemp exclusively for seeds and cultivation for two purposes, in the case of essential oils and then seeds, does not change the content of the considered compounds biochemically. This proves that Henola hemp can be cultivated in two directions at the same time and can be harvested twice because its health-promoting properties do not lose their value.

Additionally, it was found that regardless of whether hemp is grown solely for seeds or to obtain essential oils and then seeds, the type of fertilization does not create separate groups and therefore does not affect the content of phenolic acids.

Based on the conducted research, it was found that essential oils can be obtained from one plant in the summer and seeds from Henola hemp cultivation in the autumn, because such a treatment does not affect the content of the compounds in question.

## Figures and Tables

**Table 1:** Contents of fatty acids in hemp seeds (%) and coefficients of variation.

		Seeds: Henola 2									
Name of fatty acids		Fatty acid pattern	Control			Fertilizer: NPK			Fertilizer: NPK+micro		
			$\bar{x}$	±SD	V [%]	$\bar{x}$	±SD	V [%]	$\bar{x}$	±SD	V [%]
Saturated fatty acids	Myristic acid	C14:0	0.05	±0.01	20.0	0.09	±0.02	22.2	0.06	±0.03	50.0
	Palmitic acid	C16:0	6.15	±0.49	8.0	6.49	±0.21	3.2	6.64	±0.17	2.6
	Stearic acid	C18:0	2.48	±0.21	8.5	2.20	±0.10	4.5	2.38	±0.17	7.1
	Arachidic acid	C20:0	0.60	±0.07	11.7	0.54	±0.06	11.1	0.57	±0.04	7.0
	Behenic acid	C22:0	0.80	±0.06	7.5	0.70	±0.05	7.1	0.72	±0.11	15.3
	Lignoceric acid	C24:0	0.05	±0.03	60.0	0.06	±0.06	100.0	0.07	±0.05	71.4
Monounsaturated fatty acids	Palmitoleic acid	C16:1	0.14	±0.04	28.6	0.14	±0.02	14.3	0.16	±0.01	6.3
	Oleic acid	C18:1	11.58	±0.35	3.0	12.38	±0.28	2.3	11.71	±0.44	3.8
	Gondolaic acid	C20:1	1.07	±0.21	19.6	0.92	±0.78	84.8	1.32	±1.11	84.1
	Erucic acid	C22:1	0.16	±0.02	12.5	0.16	±0.02	12.5	0.15	±0.03	20.0
Polyunsaturated fatty acids	Linoleic acid	C18:2 n−6	54.90	±1.63	3.0	56.79	±0.94	1.7	54.63	±1.19	2.2
	γ-linolenic acid GLA	C18:3 n−6	0.75	±0.11	14.7	0.62	±0.02	3.2	0.74	±0.14	18.9
	α-linolenic acid	C18:3 n−3	20.82	±0.41	2.0	21.26	±0.41	1.9	21.88	±0.64	2.9
	Stearidonic acid	C18:4 n−3	0.22	±0.06	27.3	0.33	±0.03	9.1	0.35	±0.06	17.1
	Eicosadienoic acid	C20:2	0.07	±0.01	14.3	0.06	±0.03	50.0	0.06	±0.03	50.0
		Seeds: Henola 1									
Name of fatty acids		Fatty acid pattern	Control			Fertilizer: NPK			Fertilizer:NPK+micro		
			*x*	±*SD*	V [%]	*x*	±*SD*	V [%]	$\bar{x}$	±SD	V [%]
Saturated fatty acids	Myristic acid	C14:0	0.05	±0.02	40.0	0.07	±0.04	57.1	0.07	±0.03	42.9
	Palmitic acid	C16:0	6.36	±0.07	1.1	6.32	±0.20	3.2	6.51	±0.22	3.4
	Stearic acid	C18:0	2.38	±0.18	7.6	2.38	±0.02	0.8	2.41	±0.14	5.8
	Arachidic acid	C20:0	0.65	±0.04	6.2	0.52	±0.08	15.4	0.58	±0.12	20.7
	Behenic acid	C22:0	0.73	±0.11	15.1	0.69	±0.06	8.7	0.67	±0.09	13.4
	Lignoceric acid	C24:0	0.12	±0.08	66.7	0.12	±0.03	25.0	0.08	±0.04	50.0
Monounsaturated fatty acids	Palmitoleic acid	C16:1	0.15	±0.01	6.7	0.14	±0.01	7.1	0.14	±0.03	21.4
	Oleic acid	C18:1	11.45	±0.35	3.1	11.53	±0.22	1.9	11.47	±0.28	2.4
	Gondolaic acid	C20:1	1.84	±0.89	48.4	0.78	±0.91	116.7	2.93	±0.96	32.8
	Erucic acid	C22:1	0.19	±0.03	15.8	0.14	±0.02	14.3	0.15	±0.02	13.3
Polyunsaturated fatty acids	Linoleic acid	C18:2 n−6	55.35	±0.67	1.2	55.83	±0.38	0.7	54.97	±0.63	1.1
	γ-linolenic acid GLA	C18:3 n−6	0.57	±0.13	22.8	0.73	±0.15	20.5	0.76	±0.06	7.9
	α-linolenic acid	C18:3 n−3	20.41	±0.32	1.6	20.31	±0.28	1.4	21.44	±0.58	2.7
	Stearidonic acid	C18:4 n−3	0.37	±0.06	16.2	0.34	±0.08	23.5	0.42	±0.04	9.5
	Eicosadienoic acid	C20:2	0.06	±0.02	33.3	0.06	±0.01	16.7	0.05	±0.01	20.0

SD—standard deviation, *p* = 0.05.

**Table 2:** Contents of phenolic acids in hemp seeds (µg/100 g).

Seeds: Henola 2						
	Control		Fertilizer: NPK		Fertilizer:NPK+micro	
	$\bar{x}$	±SD	$\bar{x}$	±SD	$\bar{x}$	±SD
Phenolic acids $\left(\mu g/100\;g\right)$						
p-hydroxybenzoic acid	6.27	±0.43	6.65	±1.98	7.95	±4.13
vanillic	1.39	±0.08	1.47	±0.20	1.52	±0.15
caffeic	0.12	±0.02	0.15	±0.04	0.15	±0.04
p-coumaric	1.46	±0.31	1.56	±0.51	1.37	±0.18
sinapic	4.29	±1.47	3.64	±0.32	3.25	±0.28
ferulic	1.62	±0.81	1.68	±0.29	1.64	±0.52
catechins (sum)	647.62	±27.02	668.64	±43.74	649.00	±49.85
syringic	69.01	±6.61	69.69	±6.21	74.37	±3.77
benzoic	28.65	±5.69	34.59	±0.73	35.18	±3.76
Seeds: Henola 1						
	Control		Fertilizer: NPK		Fertilizer:NPK+micro	
	$\bar{x}$	±SD	$\bar{x}$	±SD	$\bar{x}$	±SD
Phenolic acids $\left(\mu g/100\;g\right)$						
p-hydroxybenzoic acid	7.32	±3.71	7.87	±3.86	6.23	±2.13
vanillic	1.41	±0.15	1.63	±0.42	1.56	±1.15
caffeic	0.17	±0.10	0.15	±0.08	0.11	±0.05
p-coumaric	2.29	±1.35	1.91	±0.91	2.24	±1.41
sinapic	3.74	±1.17	4.68	±1.65	4.43	±1.64
ferulic	2.13	±0.89	1.77	±0.94	1.98	±1.09
catechins (sum)	649.63	±38.98	707.32	±34.31	703.37	±53.55
syringic	64.68	±10.74	75.14	±1.36	71.07	±8.92
benzoic	33.57	±5.89	32.53	±3.35	39.19	±2.035

±SD—standard deviation, *p* = 0.05.

**Table 3:** Contents of flavonoids in hemp seeds (µg/100 g).

Seeds: Henola 2						
	Control		Fertilizer: NPK		Fertilizer:NPK+micro	
	$\bar{x}$	±SD	$\bar{x}$	±SD	$\bar{x}$	±SD
Flavonoids $\left(\mu g/100\;g\right)$						
rutin	0.77	±0.52	0.82	±0.09	0.73	±0.32
naringenin	863.63	±60.14	842.35	±8.40	867.00	±45.45
quercetin	17.16	±2.48	18.59	±3.90	20.34	±10.83
Seeds: Henola 1						
	Control		Fertilizer: NPK		Fertilizer:NPK+micro	
	$\bar{x}$	±SD	$\bar{x}$	±SD	$\bar{x}$	±SD
Flavonoids $\left(\mu g/100\;g\right)$						
rutin	0.85	±0.25	0.99	±0.46	0.86	±0.47
naringenin	765.60	±97.62	778.37	±71.40	783.50	±44.84
quercetin	18.21	±1.01	16.92	±0.92	12.12	±0.59

±SD—standard deviation, *p* = 0.05.

**Table 4:** Identification of volatile organic compounds in hemp seeds.

		Retention Time	Retention Index/Literary	Retention Index Actual	Odour Characteristic
MONOTERPENE	α-Pinene	6.95	936	942	Terpeny, Fruity, Sweet, Green, Woody, Pine, Citrus, Lime, Camphor
	β-Pinene	7.72	964	987	Musty, Green, Sweet, Pine, Resin, Turpentine, Woody
	β-Myrcene	7.8	991	992	Metallic, Musty, Geranium, Sweet, Fruity, Ethereal, Soapy, Lemon, Spicy, Woody
	p-cymene	8.14	1025	1013	Lemon, Fruity, Fuel-Like, Sweet, Herbal, Spicy
	Limonene	8.55	1036	1040	Licorice, Green, Citrus, Ethereal, Fruity
	Eucalyptol	8.61	1024	1043	Camphor, Minty, Sweet, Liquorices, Mentholic, Pine
	β-Ocimene	8.74	1050	1052	Herbal, Mild, Citrus, Sweet, Orange, Lemon
SESQUITERPENE	α-trans-bergamotene	14.16	1435	1424	Warm, Tea leaf
	Caryophyllene	14.38	1419	1442	Musty, Green, Spicy, Woody, Terpeny, Fruity, Sweet
	α-humulene	14.57	1458	1457	earthy, musky
	E-β-Farnesene	14.84	1456	1478	Oily, Fruity, Citrus, Woody
	Z-β-farnesene	14.9	1443	1483	Woody, Green
	α-Farnesene	15.26	1508	1514	Woody
	Selina-3,7(11)-diene	15.77	1550	1569	Woody
	Caryophyllene oxide	16.14	1589	1613	Sweet, Fruity, Sawdust, Fruity, Herbal
	Humulene-1,2-epoxide	16.34	1607	1644	Creamy, Fatty, Buttery

**Table 5:** Contents of sterols (mg/100 g) and carotenoids (mg/kg) in hemp seeds.

	Henola 2						
		Control		Fertilizer: NPK		Fertilizer: NPK + micro	
		$\bar{x}$	±SD	$\bar{x}$	±SD	$\bar{x}$	±SD
Triterpens—sterols (mg/100 g)	Campesterol	52.61	±1.03	53.99	±0.23	55.80	±1.14
	Stigmasterol	9.74	±0.32	8.71	±0.60	7.66	±0.44
	β-sitosterol	174.00	±16.72	178.67	±0.78	179.57	±9.68
	Δ5 avenasterol	15.80	±1.88	15.41	±0.61	17.92	±1.79
Tetraterpens—carotenoids (mg/100 g)	Lutein	8.26	±0.44	7.79	±0.40	8.68	±0.72
	Zeaxanthin	4.67	±0.52	5.56	±1.44	4.89	±1.42
	β–carotene	45.52	±9.49	46.84	±10.17	58.96	±10.35
	Henola 1						
		Control		Fertilizer: NPK		Fertilizer: NPK + micro	
		$\bar{x}$	±SD	$\bar{x}$	±SD	$\bar{x}$	±SD
Triterpens—sterols (mg/100 g)	Campesterol	52.10	±3.33	47.82	±7.12	49.55	±4.05
	Stigmasterol	7.37	±0.79	8.86	±1.52	8.74	±3.96
	β-sitosterol	158.87	±12.19	162.05	±15.50	179.55	±8.62
	Δ5 avenasterol	14.32	±1.05	16.11	±2.45	17.72	±0.25
Tetraterpens—carotenoids (mg/100 g)	Lutein	6.30	±3.55	8.07	±0.32	7.82	±0.58
	Zeaxanthin	4.89	±1.35	5.41	±0.39	5.02	±1.02
	β–carotene	46.74	±7.15	57.69	±9.91	63.48	±10.74
*p* = 0.05							

**Table 6:** Comparison of Henola 1 and Henola 2 distributions using the Wilcoxon test: *p*-values.

Seeds: Phenolic Acids										Seeds: Flawonoids		
	p-hydroxybenzoic	vanillic	coffee	p-coumaric	sinapic	ferulic	catechins	syringing	benzoic	rutin	naringenin	quercetin
Control	0.7	1	0.7	0.4	1	0.4	0.99	1	0.4	0.7	0.4	0.7
NPK	0.82	1	1	0.7	0.1	0.7	0.7	0.4	0.4	0.7	0.4	0.7
NPK+micro	1	1	1	0.5	0.7	1	0.7	1	0.2	1	0.1	0.3

## Data Availability

Data are contained within the article.
